# (*E*)-3-(9-Anthr­yl)-1-(4-fluoro­phen­yl)-2-(4-nitro-1*H*-imidazol-1-yl)prop-2-en-1-one

**DOI:** 10.1107/S1600536809055524

**Published:** 2010-01-09

**Authors:** Xiao-Ling Wang, Guang-Zhou Wang, Rong-Xia Geng, Cheng-He Zhou

**Affiliations:** a School of Chemistry and Chemical Engineering, Southwest University, Chongqing 400715, People’s Republic of China

## Abstract

In the title compound, C_26_H_16_FN_3_O_3_, the dihedral angle between the anthryl and fluoro­phenyl groups is 37.8 (1)°. With respect to the imidazolyl group, the twist angles between the imidazolyl group and the anthryl unit and between the imidazoly group and the fluoro­phenyl group are 64.4 (1) and 74.5 (1)°, respectively.

## Related literature

For general background to chalcone derivatives, see: Detsi *et al.* (2009 ▶). For the synthesis, see: Erhardt *et al.* (1985 ▶); Kranz *et al.* (1980 ▶). For related structures, see: Lu *et al.* (2009 ▶); Wang *et al.* (2009 ▶). For a comment on the mol­ecular shape, see: Hou *et al.* (2009 ▶).
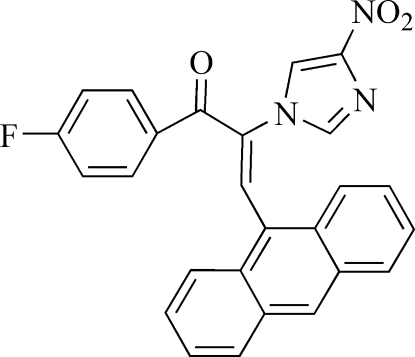


## Experimental

### 

#### Crystal data

C_26_H_16_FN_3_O_3_
*M* *_r_* = 437.42
Triclinic, 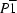

*a* = 9.3362 (6) Å
*b* = 10.9587 (6) Å
*c* = 11.6018 (5) Å
α = 70.371 (5)°
β = 88.062 (4)°
γ = 66.781 (6)°
*V* = 1020.78 (10) Å^3^
*Z* = 2
Mo *K*α radiation
μ = 0.10 mm^−1^
*T* = 173 K
0.49 × 0.41 × 0.30 mm

#### Data collection

Oxford Diffraction Xcaliber diffractometer
Absorption correction: multi-scan (*CrysAlis RED*; Oxford Diffraction, 2009 ▶) *T*_min_ = 0.951, *T*_max_ = 0.970
8826 measured reflections
4374 independent reflections
3193 reflections with *I* > 2σ(*I*)
*R*_int_ = 0.020

#### Refinement

*R*[*F* ^2^ > 2σ(*F* ^2^)] = 0.036
*wR*(*F* ^2^) = 0.090
*S* = 1.01
4374 reflections
298 parameters
H-atom parameters constrained
Δρ_max_ = 0.22 e Å^−3^
Δρ_min_ = −0.19 e Å^−3^

### 

Data collection: *CrysAlis PRO* (Oxford Diffraction, 2009 ▶); cell refinement: *CrysAlis PRO*; data reduction: *CrysAlis PRO*; program(s) used to solve structure: *SHELXS97* (Sheldrick, 2008 ▶); program(s) used to refine structure: *SHELXL97* (Sheldrick, 2008 ▶); molecular graphics: *SHELXTL* (Sheldrick, 2008 ▶); software used to prepare material for publication: *SHELXTL*.

## Supplementary Material

Crystal structure: contains datablocks I, global. DOI: 10.1107/S1600536809055524/ng2707sup1.cif

Structure factors: contains datablocks I. DOI: 10.1107/S1600536809055524/ng2707Isup2.hkl

Additional supplementary materials:  crystallographic information; 3D view; checkCIF report

## Figures and Tables

**Fig. 1.**
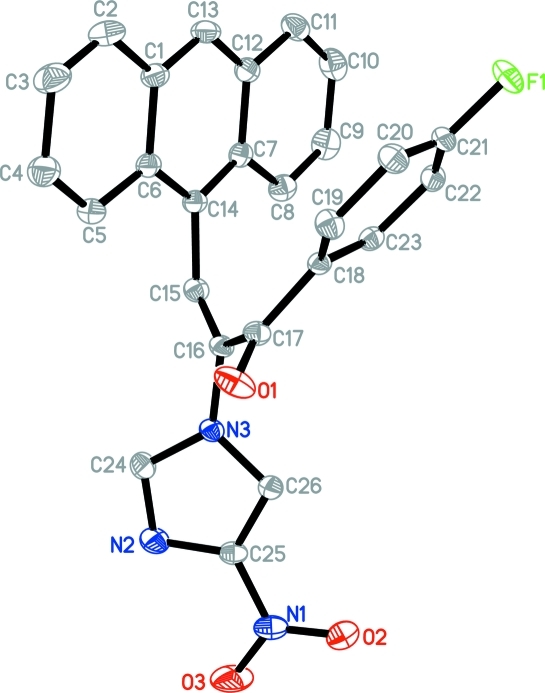
The molecular structure of (I), showing theatom-numbering scheme. Displacement ellipsoids are drawn at the 50% probability level.

**Fig. 2.**
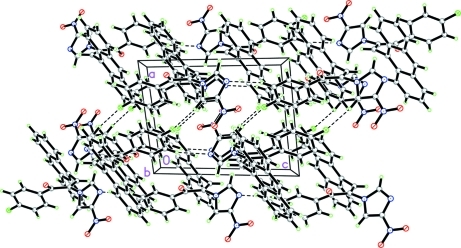
Part of the CrystalStructure of (I), showing the formation of the three-dimensional network.

## supplementary crystallographic information

### Comment

Chalcones (1,3-diaryl-2-propen-1-ones) are flavonoid and isoflavnoid precursors which are abundant in edible plants and display wide biological activities such as antioxidant, antibacterial, antileishmanial, anticancer, antiangiogenic, anti-infective and anti-inflammatory activities. Chalcone derivatives have received much attention due to their relatively simple structures, and wide variety of biological activities (Detsi *et al.* 2009). A series of chalcone derivatives containing imidazole ring have been synthesized and crystal structures of some of them have been reported (Lu *et al.* 2009; Wang *et al.* 2009). We report here the structure of the title compound (I).

The title compound (I), C_26_H_16_FN_3_O_3_, shows an organic-clip-shaped motif (Hou *et al.* 2009). The ringent dihedral angle between the anthryl unit and the fluorophenyl group is 37.80°. The imidazolyl group can be seen as the handle of organic clip, and the dihedral angles between the imidazolyl group and the anthryl unit or the fluorophenyl group are 64.40° and 74.51° respectively. In the solid state, the compound (I) is stabilized by weak intermolecule C—H···O and C—H···F hydrogen bonds generating an infinite two-dimensional network.

### Experimental

Compound (I) was synthesized according to the procedure of Erhardt *et al.* (1985) and Kranz *et al.* (1980). Single crystals (I) suitable for X-ray analysis were grown in dichloromethane by slow evaporation at room temperature.

### Refinement

Hydrogen atoms were placed in calculated positions with C—H = 0.95Å (aromatic ring) with *U*iso(H) = 1.2*U*eq(C).

### Crystal data

**Table:**

C_26_H_16_FN_3_O_3_	*Z* = 2
*M**_r_* = 437.42	*F*(000) = 452
Triclinic, *P*1	*D*_x_ = 1.423 Mg m^−^^3^
Hall symbol: -P 1	Mo *K*α radiation, λ = 0.71073 Å
*a* = 9.3362 (6) Å	Cell parameters from 8826 reflections
*b* = 10.9587 (6) Å	θ = 2.8–27.0°
*c* = 11.6018 (5) Å	µ = 0.10 mm^−^^1^
α = 70.371 (5)°	*T* = 173 K
β = 88.062 (4)°	Block, yellow
γ = 66.781 (6)°	0.49 × 0.41 × 0.30 mm
*V* = 1020.78 (10) Å^3^	

### Data collection

**Table:**

Oxford Diffraction Xcaliber diffractometer	4374 independent reflections
Radiation source: fine-focus sealed tube	3193 reflections with *I* > 2σ(*I*)
graphite	*R*_int_ = 0.020
Detector resolution: 0.01 pixels mm^-1^	θ_max_ = 27.0°, θ_min_ = 2.8°
ω scans	*h* = −11→11
Absorption correction: multi-scan (*CrysAlis RED*; Oxford Diffraction, 2009)	*k* = −13→12
*T*_min_ = 0.951, *T*_max_ = 0.970	*l* = −14→14
8826 measured reflections	

### Refinement

**Table:**

Refinement on *F*^2^	Primary atom site location: structure-invariant direct methods
Least-squares matrix: full	Secondary atom site location: difference Fourier map
*R*[*F*^2^ > 2σ(*F*^2^)] = 0.036	Hydrogen site location: inferred from neighbouring sites
*wR*(*F*^2^) = 0.090	H-atom parameters constrained
*S* = 1.01	*w* = 1/[σ^2^(*F*_o_^2^) + (0.0501*P*)^2^] where *P* = (*F*_o_^2^ + 2*F*_c_^2^)/3
4374 reflections	(Δ/σ)_max_ = 0.001
298 parameters	Δρ_max_ = 0.22 e Å^−^^3^
0 restraints	Δρ_min_ = −0.19 e Å^−^^3^

### Special details

**Table:**

Geometry. All e.s.d.'s (except the e.s.d. in the dihedral angle between two l.s. planes) are estimated using the full covariance matrix. The cell e.s.d.'s are taken into account individually in the estimation of e.s.d.'s in distances, angles and torsion angles; correlations between e.s.d.'s in cell parameters are only used when they are defined by crystal symmetry. An approximate (isotropic) treatment of cell e.s.d.'s is used for estimating e.s.d.'s involving l.s. planes.
Refinement. Refinement of *F*^2^ against ALL reflections. The weighted *R*-factor *wR* and goodness of fit *S* are based on *F*^2^, conventional *R*-factors *R* are based on *F*, with *F* set to zero for negative *F*^2^. The threshold expression of *F*^2^ > σ(*F*^2^) is used only for calculating *R*-factors(gt) *etc*. and is not relevant to the choice of reflections for refinement. *R*-factors based on *F*^2^ are statistically about twice as large as those based on *F*, and *R*- factors based on ALL data will be even larger.

### Fractional atomic coordinates and isotropic or equivalent isotropic displacement parameters (Å^2^)

**Table:**

	*x*	*y*	*z*	*U*_iso_*/*U*_eq_	
F1	0.40315 (10)	0.15681 (10)	1.18206 (7)	0.0560 (3)	
O1	0.12744 (13)	−0.02448 (11)	0.81580 (8)	0.0455 (3)	
N3	0.19905 (12)	0.08673 (11)	0.58002 (8)	0.0261 (2)	
C15	0.10394 (14)	0.30540 (13)	0.61663 (11)	0.0282 (3)	
H15A	0.1064	0.3481	0.5309	0.034*	
C17	0.16584 (14)	0.07397 (13)	0.79428 (10)	0.0275 (3)	
C23	0.33391 (14)	0.16396 (13)	0.87665 (11)	0.0270 (3)	
H23A	0.3671	0.1929	0.7975	0.032*	
C14	0.04204 (14)	0.40033 (13)	0.68792 (10)	0.0272 (3)	
C6	−0.08868 (15)	0.40198 (13)	0.75307 (10)	0.0282 (3)	
C18	0.22475 (14)	0.10459 (12)	0.89378 (10)	0.0244 (3)	
C7	0.11980 (15)	0.48598 (13)	0.69277 (11)	0.0291 (3)	
C22	0.39456 (15)	0.18126 (13)	0.97408 (12)	0.0333 (3)	
H22A	0.4704	0.2207	0.9636	0.040*	
N2	0.18404 (14)	0.02358 (13)	0.41956 (9)	0.0390 (3)	
C19	0.17599 (15)	0.06337 (14)	1.01033 (10)	0.0323 (3)	
H19A	0.1023	0.0216	1.0226	0.039*	
C12	0.07161 (15)	0.56901 (13)	0.77072 (11)	0.0326 (3)	
C16	0.15658 (14)	0.16506 (13)	0.66238 (10)	0.0255 (3)	
C26	0.32395 (15)	−0.05927 (13)	0.49025 (10)	0.0295 (3)	
O2	0.56329 (12)	−0.24366 (12)	0.53163 (10)	0.0525 (3)	
C8	0.25072 (16)	0.48911 (14)	0.62676 (12)	0.0368 (3)	
H8A	0.2838	0.4365	0.5731	0.044*	
C1	−0.13552 (15)	0.48673 (14)	0.83035 (11)	0.0330 (3)	
C20	0.23331 (16)	0.08250 (15)	1.10757 (11)	0.0368 (3)	
H20A	0.1986	0.0568	1.1866	0.044*	
C21	0.34228 (15)	0.13994 (14)	1.08624 (11)	0.0346 (3)	
O3	0.41958 (15)	−0.18717 (12)	0.36400 (9)	0.0656 (4)	
C13	−0.05363 (16)	0.56575 (14)	0.83786 (12)	0.0358 (3)	
H13A	−0.0842	0.6195	0.8907	0.043*	
C5	−0.17915 (15)	0.32560 (14)	0.74550 (12)	0.0343 (3)	
H5A	−0.1529	0.2715	0.6929	0.041*	
C2	−0.26464 (17)	0.48480 (16)	0.89925 (13)	0.0437 (4)	
H2A	−0.2949	0.5386	0.9521	0.052*	
C11	0.15633 (17)	0.64982 (15)	0.77975 (12)	0.0405 (3)	
H11A	0.1242	0.7063	0.8303	0.049*	
C9	0.32888 (18)	0.56622 (15)	0.63942 (13)	0.0450 (4)	
H9A	0.4165	0.5659	0.5953	0.054*	
C4	−0.30200 (17)	0.32892 (16)	0.81208 (13)	0.0433 (4)	
H4A	−0.3604	0.2770	0.8057	0.052*	
C10	0.28170 (19)	0.64686 (16)	0.71722 (13)	0.0472 (4)	
H10A	0.3384	0.6994	0.7257	0.057*	
C3	−0.34423 (18)	0.40905 (17)	0.89124 (14)	0.0484 (4)	
H3A	−0.4292	0.4089	0.9387	0.058*	
N1	0.44252 (15)	−0.17030 (13)	0.45944 (10)	0.0389 (3)	
C25	0.33644 (15)	−0.02437 (13)	0.58946 (11)	0.0294 (3)	
H25A	0.4225	−0.0681	0.6519	0.035*	
C24	0.11069 (16)	0.11104 (15)	0.47696 (11)	0.0357 (3)	
H24A	0.0077	0.1832	0.4500	0.043*	

### Atomic displacement parameters (Å^2^)

**Table:**

	*U*^11^	*U*^22^	*U*^33^	*U*^12^	*U*^13^	*U*^23^
F1	0.0625 (6)	0.0621 (6)	0.0440 (5)	−0.0166 (5)	−0.0174 (4)	−0.0276 (5)
O1	0.0783 (8)	0.0471 (6)	0.0291 (5)	−0.0426 (6)	0.0100 (5)	−0.0145 (5)
N3	0.0312 (6)	0.0287 (6)	0.0189 (5)	−0.0111 (5)	0.0027 (4)	−0.0102 (4)
C15	0.0326 (7)	0.0304 (7)	0.0205 (6)	−0.0121 (6)	0.0030 (5)	−0.0088 (5)
C17	0.0327 (7)	0.0276 (7)	0.0239 (6)	−0.0126 (6)	0.0054 (5)	−0.0108 (5)
C23	0.0278 (6)	0.0230 (6)	0.0263 (6)	−0.0074 (5)	0.0035 (5)	−0.0077 (5)
C14	0.0313 (7)	0.0220 (6)	0.0214 (6)	−0.0051 (5)	−0.0018 (5)	−0.0057 (5)
C6	0.0308 (7)	0.0245 (6)	0.0234 (6)	−0.0056 (6)	−0.0015 (5)	−0.0075 (5)
C18	0.0259 (6)	0.0228 (6)	0.0205 (6)	−0.0052 (5)	0.0008 (5)	−0.0083 (5)
C7	0.0330 (7)	0.0205 (6)	0.0246 (6)	−0.0057 (6)	−0.0036 (5)	−0.0026 (5)
C22	0.0286 (7)	0.0277 (7)	0.0419 (7)	−0.0081 (6)	−0.0036 (6)	−0.0135 (6)
N2	0.0484 (7)	0.0445 (7)	0.0244 (5)	−0.0142 (6)	0.0014 (5)	−0.0177 (5)
C19	0.0353 (7)	0.0393 (8)	0.0243 (6)	−0.0178 (6)	0.0060 (5)	−0.0107 (6)
C12	0.0377 (7)	0.0225 (6)	0.0302 (6)	−0.0066 (6)	−0.0049 (6)	−0.0065 (5)
C16	0.0290 (6)	0.0291 (7)	0.0209 (6)	−0.0117 (6)	0.0044 (5)	−0.0122 (5)
C26	0.0372 (7)	0.0302 (7)	0.0245 (6)	−0.0151 (6)	0.0099 (5)	−0.0127 (5)
O2	0.0431 (6)	0.0485 (7)	0.0645 (7)	−0.0085 (6)	0.0074 (6)	−0.0305 (6)
C8	0.0407 (8)	0.0285 (7)	0.0353 (7)	−0.0119 (6)	0.0036 (6)	−0.0069 (6)
C1	0.0354 (7)	0.0281 (7)	0.0298 (6)	−0.0055 (6)	0.0014 (6)	−0.0121 (6)
C20	0.0419 (8)	0.0438 (8)	0.0208 (6)	−0.0119 (7)	0.0033 (6)	−0.0131 (6)
C21	0.0353 (7)	0.0334 (7)	0.0298 (7)	−0.0028 (6)	−0.0101 (6)	−0.0166 (6)
O3	0.0953 (9)	0.0581 (7)	0.0393 (6)	−0.0133 (7)	0.0112 (6)	−0.0341 (6)
C13	0.0430 (8)	0.0282 (7)	0.0336 (7)	−0.0073 (6)	0.0005 (6)	−0.0160 (6)
C5	0.0349 (7)	0.0352 (7)	0.0345 (7)	−0.0116 (6)	0.0036 (6)	−0.0173 (6)
C2	0.0483 (9)	0.0418 (8)	0.0443 (8)	−0.0138 (7)	0.0155 (7)	−0.0254 (7)
C11	0.0521 (9)	0.0298 (7)	0.0375 (7)	−0.0148 (7)	−0.0069 (7)	−0.0100 (6)
C9	0.0450 (9)	0.0385 (8)	0.0485 (8)	−0.0202 (7)	0.0043 (7)	−0.0077 (7)
C4	0.0401 (8)	0.0457 (9)	0.0535 (9)	−0.0208 (7)	0.0118 (7)	−0.0253 (7)
C10	0.0545 (10)	0.0359 (8)	0.0505 (9)	−0.0245 (8)	−0.0082 (8)	−0.0058 (7)
C3	0.0439 (9)	0.0539 (10)	0.0549 (9)	−0.0196 (8)	0.0226 (7)	−0.0301 (8)
N1	0.0520 (8)	0.0349 (7)	0.0357 (6)	−0.0196 (6)	0.0167 (6)	−0.0184 (6)
C25	0.0311 (7)	0.0282 (7)	0.0286 (6)	−0.0099 (6)	0.0008 (5)	−0.0120 (6)
C24	0.0371 (8)	0.0422 (8)	0.0240 (6)	−0.0100 (7)	−0.0023 (6)	−0.0140 (6)

### Geometric parameters (Å, °)

**Table:**

F1—C21	1.3626 (13)	C12—C11	1.4288 (18)
O1—C17	1.2135 (14)	C26—C25	1.3508 (15)
N3—C25	1.3521 (16)	C26—N1	1.4272 (17)
N3—C24	1.3641 (15)	O2—N1	1.2312 (15)
N3—C16	1.4367 (14)	C8—C9	1.3583 (18)
C15—C16	1.3273 (17)	C8—H8A	0.9500
C15—C14	1.4739 (16)	C1—C13	1.3854 (18)
C15—H15A	0.9500	C1—C2	1.4270 (19)
C17—C18	1.4821 (15)	C20—C21	1.3696 (19)
C17—C16	1.5034 (16)	C20—H20A	0.9500
C23—C22	1.3806 (16)	O3—N1	1.2210 (13)
C23—C18	1.3869 (16)	C13—H13A	0.9500
C23—H23A	0.9500	C5—C4	1.3563 (18)
C14—C7	1.4095 (17)	C5—H5A	0.9500
C14—C6	1.4109 (17)	C2—C3	1.339 (2)
C6—C5	1.4260 (17)	C2—H2A	0.9500
C6—C1	1.4389 (16)	C11—C10	1.351 (2)
C18—C19	1.3963 (16)	C11—H11A	0.9500
C7—C8	1.4262 (18)	C9—C10	1.411 (2)
C7—C12	1.4319 (17)	C9—H9A	0.9500
C22—C21	1.3682 (18)	C4—C3	1.4192 (19)
C22—H22A	0.9500	C4—H4A	0.9500
N2—C24	1.3082 (16)	C10—H10A	0.9500
N2—C26	1.3565 (16)	C3—H3A	0.9500
C19—C20	1.3752 (16)	C25—H25A	0.9500
C19—H19A	0.9500	C24—H24A	0.9500
C12—C13	1.3885 (18)		
			
C25—N3—C24	106.98 (10)	C13—C1—C2	121.72 (11)
C25—N3—C16	126.08 (10)	C13—C1—C6	119.74 (12)
C24—N3—C16	126.93 (11)	C2—C1—C6	118.54 (12)
C16—C15—C14	125.33 (11)	C21—C20—C19	117.49 (12)
C16—C15—H15A	117.3	C21—C20—H20A	121.3
C14—C15—H15A	117.3	C19—C20—H20A	121.3
O1—C17—C18	121.96 (11)	F1—C21—C22	117.71 (12)
O1—C17—C16	118.70 (10)	F1—C21—C20	118.41 (11)
C18—C17—C16	119.33 (10)	C22—C21—C20	123.89 (11)
C22—C23—C18	120.32 (11)	C1—C13—C12	122.18 (11)
C22—C23—H23A	119.8	C1—C13—H13A	118.9
C18—C23—H23A	119.8	C12—C13—H13A	118.9
C7—C14—C6	120.79 (10)	C4—C5—C6	121.13 (12)
C7—C14—C15	118.37 (11)	C4—C5—H5A	119.4
C6—C14—C15	120.80 (11)	C6—C5—H5A	119.4
C14—C6—C5	123.66 (11)	C3—C2—C1	121.60 (12)
C14—C6—C1	118.58 (11)	C3—C2—H2A	119.2
C5—C6—C1	117.76 (11)	C1—C2—H2A	119.2
C23—C18—C19	119.36 (11)	C10—C11—C12	120.76 (13)
C23—C18—C17	122.07 (10)	C10—C11—H11A	119.6
C19—C18—C17	118.43 (11)	C12—C11—H11A	119.6
C14—C7—C8	122.54 (11)	C8—C9—C10	120.93 (14)
C14—C7—C12	119.57 (11)	C8—C9—H9A	119.5
C8—C7—C12	117.84 (11)	C10—C9—H9A	119.5
C21—C22—C23	118.05 (12)	C5—C4—C3	120.74 (13)
C21—C22—H22A	121.0	C5—C4—H4A	119.6
C23—C22—H22A	121.0	C3—C4—H4A	119.6
C24—N2—C26	103.31 (10)	C11—C10—C9	120.38 (13)
C20—C19—C18	120.87 (12)	C11—C10—H10A	119.8
C20—C19—H19A	119.6	C9—C10—H10A	119.8
C18—C19—H19A	119.6	C2—C3—C4	120.17 (13)
C13—C12—C11	121.85 (12)	C2—C3—H3A	119.9
C13—C12—C7	119.02 (11)	C4—C3—H3A	119.9
C11—C12—C7	119.10 (12)	O3—N1—O2	123.78 (12)
C15—C16—N3	119.29 (10)	O3—N1—C26	118.88 (12)
C15—C16—C17	127.06 (10)	O2—N1—C26	117.34 (11)
N3—C16—C17	113.45 (10)	C26—C25—N3	104.56 (11)
C25—C26—N2	112.89 (11)	C26—C25—H25A	127.7
C25—C26—N1	125.20 (12)	N3—C25—H25A	127.7
N2—C26—N1	121.91 (11)	N2—C24—N3	112.26 (12)
C9—C8—C7	120.97 (12)	N2—C24—H24A	123.9
C9—C8—H8A	119.5	N3—C24—H24A	123.9
C7—C8—H8A	119.5		
